# Neutrophils Are Essential As A Source Of Il-17 In The Effector Phase Of Arthritis

**DOI:** 10.1371/journal.pone.0062231

**Published:** 2013-05-06

**Authors:** Masaki Katayama, Koichiro Ohmura, Naoichiro Yukawa, Chikashi Terao, Motomu Hashimoto, Hajime Yoshifuji, Daisuke Kawabata, Takao Fujii, Yoichiro Iwakura, Tsuneyo Mimori

**Affiliations:** 1 Department of Rheumatology and Clinical Immunology, Graduate School of Medicine, Kyoto University, Kyoto, Japan; 2 Center for Genetic Medicine, Graduate School of Medicine, Kyoto University, Kyoto, Japan; 3 Department of the Control for Rheumatic Diseases, Graduate School of Medicine, Kyoto University, Kyoto, Japan; 4 Center for Experimental Medicine and Systems Biology, Institute of Medical Science, University of Tokyo, Tokyo, Japan; Blood Systems Research Institute, United States of America

## Abstract

**Objective:**

Th17 has been shown to have a pivotal role in the development of arthritis. However, the role of IL-17 in the T cell-independent effector phase has not fully been examined. We investigated whether IL-17 is involved in the effector phase of arthritis by using K/BxN serum-induced arthritis model.

**Methods:**

K/BxN serum was transferred into IL-17 knockout (KO) mice, SCID mice and their control mice, and arthritis was evaluated over time. In order to clarify the source of IL-17 in the effector phase, neutrophils or CD4+ T cells collected from IL-17 KO or control mice were injected into IL-17 KO recipient mice together with K/BxN serum. To examine if neutrophils secrete IL-17 upon stimulation, neutrophils were stimulated with immune complex in vitro and IL-17 in the supernatant was measured by ELISA.

**Results:**

K/BxN serum-induced arthritis was much less severe in IL-17 KO mice than in WT mice. Since K/BxN serum-transferred SCID mice developed severe arthritis with high serum IL-17 concentration, we speculated neutrophils are the responsible player as an IL-17 source. When wild type (WT) but not IL-17 KO neutrophils were co-injected with K/BxN serum into IL-17 KO mice, arthritis was exacerbated, whereas co-injection of WT CD4+ T cells had no effect. In vitro, stimulation of neutrophils with immune complexcaused IL-17 secretion.

**Conclusions:**

Neutrophils are essential as a source of IL-17 in the effector phase of arthritis. The trigger of secreting IL-17 from neutrophils may be immune complex.

## Introduction

Rheumatoid arthritis (RA) is a chronic inflammatory disease characterized by synovitis leading to destruction of articular cartilage and bone. The pathophysiology of RA is still unclear, but recently the important role of IL-17-producing T cells (Th17) has been highlighted in arthritis development in human and several mouse models. IL-17 (especially IL-17A) is a proinflammatory cytokine that is thought to contribute to the inflammation, cartilage destruction and bone erosion in RA. IL-17 is upregulated in the synovium and the synovial fluids of RA patients 1. IL-17 induces fibroblasts, endothelial cells or macrophages to secrete IL-6, TNFα and IL-1 2,3,4. IL-17 can synergize with IL-1 and TNFα, although it may also have direct pathological effects. In experimental arthritis models, the critical role of IL-17 has been clearly demonstrated. Spontaneous development of destructive arthritis in mice deficient in IL-1 receptor antagonist was completely abrogated in the absence of IL-17 5. Collagen-induced arthritis 3 was also apparently suppressed in IL-17 knock out (KO) mice and was prevented by anti-IL-17 antibody treatment 6.

K/BxN mouse is a KRN T cell receptor transgenic mouse crossed with NOD mouse, and develops severe arthritis similar to RA 7. KRN T cells recognize glycolytic enzyme glucose-6-phosphatase isomerase (GPI), and the autoantibodies against GPI cause arthritis. Transfer of K/BxN arthritic serum or purified anti-GPI antibodies into normal mice induces arthritis similar to K/BxN mice 8. In the K/BxN model, T cells and B cells are required for inducing arthritis, but once the anti-GPI antibody is generated, arthritis can develop without lymphocytes 9. K/BxN serum-transfer arthritis is thus a useful model to analyze the effector phase of arthritis. The innate immune system including neutrophils 10, mast cells 11, Fcγ receptor and C5a 12 has been shown to be essential in this arthritis development. Although IL-1 and TNFα, but not IL-6, were shown to be important in this arthritis model 13, it has not been clear whether IL-17 works in the effector phase of arthritis, regardless of the findings that non-T cells produce IL-17. In the present study, we tested whether IL-17 is involved in K/BxN serum-induced arthritis using IL-17 KO mice and we found that IL-17 derived from neutrophils affects arthritis severity in the effector phase.

## Materials And Methods

### Mice

C57BL/6JJcl (B6), FOX CHASE SCID C.B-17/Icr-scid/scidJcl (SCID), FOX CHASE SCID C.B-17/Icr-+/+Jcl (SCID WT), NOD/SciJcl (NOD) mice were purchased from Japan Clea Inc. (Tokyo, Japan). KRN TCR transgenic (B6 background: K/B) and Cα (TCRα chain) KO mice were kindly provided by Drs. D. Mathis and C. Benoist, Harvard Medical School, Boston, MA. Fcγ receptor KO (FcR KO) mice were kindly provided by Dr. T. Takai, Tohoku University, Sendai. All mice were maintained in our animal facility under specific pathogen–free conditions. K/BxN arthritic mice were obtained by crossing K/B with NOD mice, and the sera were pooled at eight weeks old. Arthritic adult K/BxN mice were bled, and the sera collected from eight-week-old K/BxN mice were pooled. All animal procedures were approved by the Ethics Committee of Kyoto University.

### Induction Of K/bxn Serum-Induced Arthritis And Arthritis Scoring

Recipient mice were usually i.p. injected with 200 µl of K/BxN sera at days 0 and 2. In the experiments of neutrophil or CD4^+^ T cell transfer, recipient mice were i.v. injected with 200 µl of K/BxN sera at days 0 and 2. Arthritis was evaluated visually, and the swelling of each paw was scored on a scale of 0–3, where 0 = no evidence of inflammation, 1 = subtle inflammation or localized edema, 2 = easily identified swelling localized to either the dorsal or ventral surface of the paw, and 3 = swelling of all aspects of the paw. Clinical indices for all four paws were added as a composite score. Ankle thickness was measured with a caliper.

### Histological Examination

Dissected ankles were fixed in 4% neutral buffered paraformaldehyde, demineralized and stained with hematoxylin and eosin (H&E).

### Isolation Of Murine Neutrophils From Bone Marrow (bm) And Cd4^+^ T Cells From Spleen

BM neutrophils were isolated using a mouse Anti-Ly-6G MicroBead Kit (Miltenyi Biotec, Bergisch Gladbach, Germany). The purity of neutrophils was >95% as determined by May-Giemsa staining. Splenic CD4^+^ T cells were isolated using a mouse Pan T Cell Isolation Kit II (Miltenyi Biotec).

### Neutrophil Stimulation In Vitro

Soluble murine peroxidase-anti-peroxidase (mPAP) ICs consisting primarily of two horseradish peroxidase (HRP) molecules bound to three anti-peroxidase IgGs were obtained from Jackson ImmunoResearch Laboratories (Bar Harbor, HE, USA). Immunomagnetically purified neutrophils from mouse BM were incubated at a concentration of 8×10^6^/ml in RPMI 1640 medium containing 10%FCS, penicillin, or streptomycin for 3 hours, followed by culture with mPAP IC (200, 20, and 2 µg/ml), HRP (Sigma Aldrich, Saint Luis, MO, USA: 200 µg/ml), anti-HRP antibody (Thermo Fisher Scientific, Waltham, MA, USA: 200 µg/ml) or PBS for 1 hour before collecting the culture supernatant.

### Il-17 Measurements By Elisa

Mouse IL-17 Quantikine EILSA kit was purchased from R&D systems (Minneapolis, MN, USA). Cell culture supernatant and sera from arthritic mice were assayed in accordance with the manufacturer’s instructions.

### Statistical Analysis

Statistical analysis was performed using the Student’s *t-*test. Data are expressed as means ± SEM unless otherwise stated.

## Results

### Il-17 Exacerbates K/bxn Serum-Induced Arthritis

**Figure 1:**
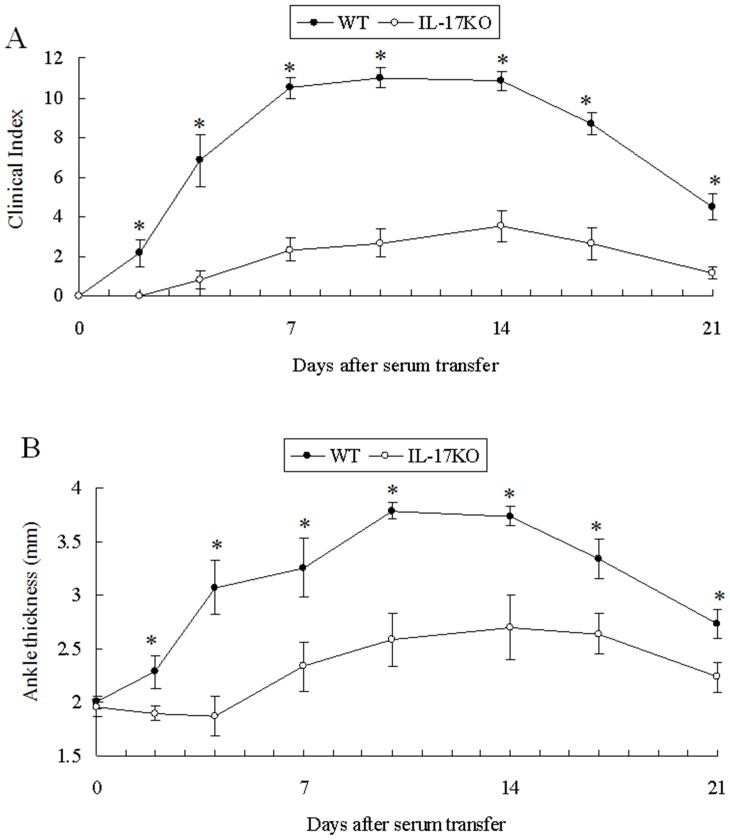
K/BxN serum-induced arthritis in IL-17 knockout mice. K/BxN sera (200 µl/body) were i.p. injected at days 0 and 2 into IL-17 KO (○) and WT mice (•) (n = 6 each). Clinical index (A) and ankle thickness (B) were monitored for 21 days. The results were from two independent experiments, both of which showed similar results. *p<0.05.

**Figure 2:**
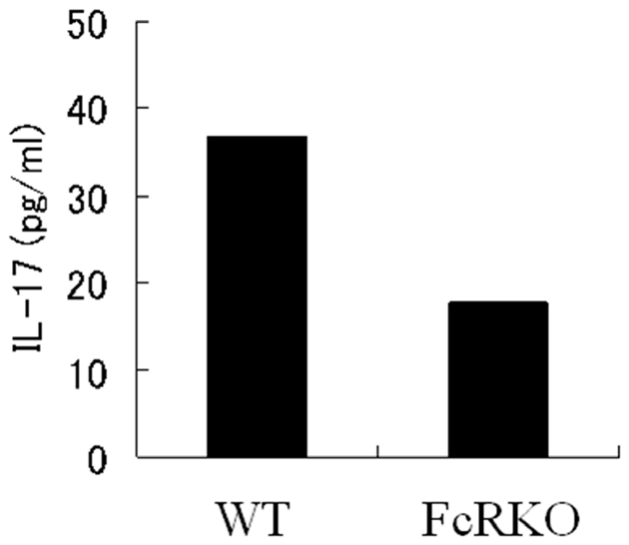
Histopathology of ankle joints from arthritic IL-17KO and WT mice. Arthritis was induced as described in the legend for figure 1 and the mice were sacrificed at days 7 and 21 after initial serum transfer. H&E stainings of ankle joints are shown (scale bar represents 100 µm).

To clarify whether IL-17 has any roles in the effector phase of arthritis, we induced K/BxN serum-induced arthritis in IL-17 KO and WT B6 mice. We assessed the clinical index and ankle thickness over time. Interestingly, the severity of arthritis in IL-17 KO mice was much milder than that in WT mice (Fig. 1). Histological examination of ankle joints also revealed that the inflammation, bone and cartilage destruction of serum induced arthritis were much less in IL-17 KO mice than in WT mice (Fig. 2). These results clearly indicate that IL-17 has pivotal roles in aggravating arthritis in the effector phase.

### Cd4+t Cells Are Not The Source Of Il-17 In Arthritis Effector Phase

**Figure 3:**
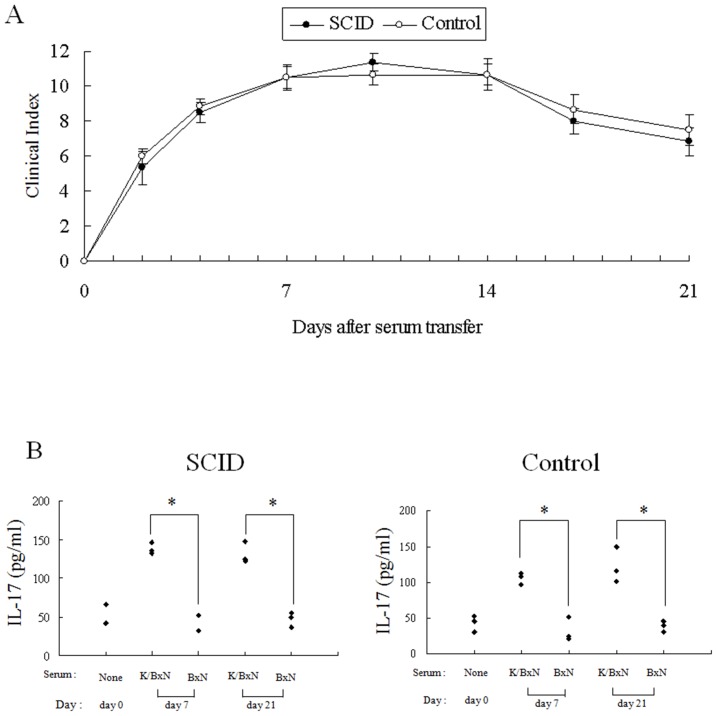
Arthritis severity and IL-17 in the sera of SCID or control mice which received K/BxN or BxN sera. K/BxN arthritic sera or BxN control sera were injected into SCID (C.B-17/Icr-scid/scid) and corresponding control mice (C.B-17/Icr-+/+) at days 0 and 2. (A) Clinical Index was monitored for 21 days. (B) Blood was sampled at days 0, 7 and 21 and IL-17 concentrations in the sera of each mouse were measured by EILSA. *p<0.05.

We hypothesized that T cells were not the source of IL-17 in arthritis effector phase since it was reported that T cells are not essential in this phase. To confirm this, we injected K/BxN arthritic sera or BxN control sera into SCID and control mice (C.B-17/Icr-+/+). We assessed their clinical index and ankle thickness over time, and measured their serum concentration of IL-17 at days 7 and 21. The arthritis induced in SCID mice was as severe as that in control mice (Fig. 3A), and the concentrations of serum IL-17 in the SCID mice were similar to those in the control mice (Fig. 3B). In contrast, in both SCID and control mice, the concentrations of serum IL-17 in BxN serum-injected non-arthritic mice were significantly lower than those in K/BxN serum-induced arthritic mice (Fig. 3B). Since the concentration of IL-17 in the injected K/BxN serum was ∼50 pg/ml (data not shown) and that of K/BxN serum-induced SCID mice was ∼140 pg/ml (Fig. 3B), it was evident that the serum IL-17 in the K/BxN serum-induced SCID mice was not derived from K/BxN serum itself but from non-T non-B inflammatory cells.

### Neutrophils Exacerbate Arthritis Via Il-17 In The Effector Phase

**Figure 4:**
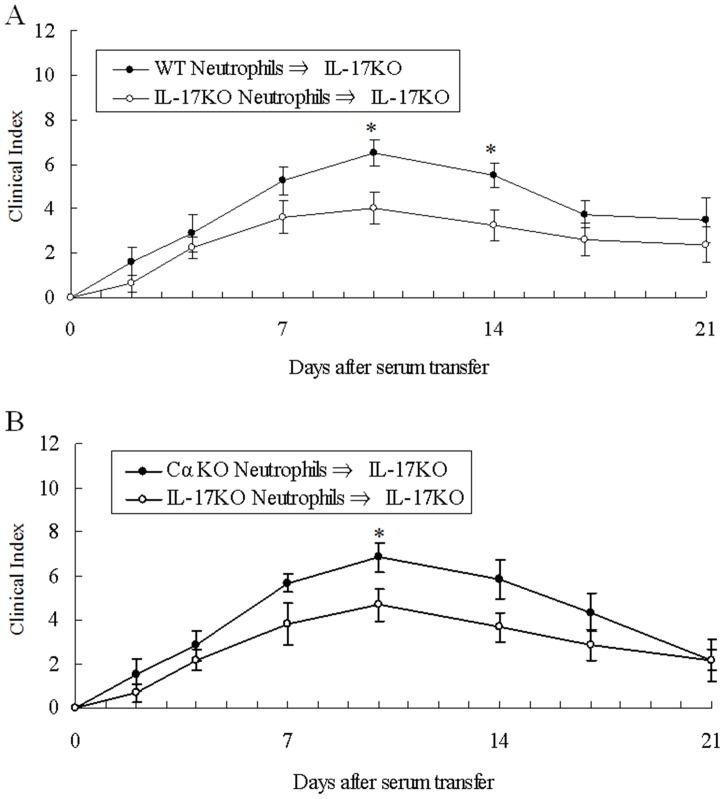
IL-17 from neutrophils aggravates K/BxN serum-induced arthritis. (A) Neutrophils (2×10^6^/body) collected from bone marrow of IL-17 KO (○) or WT (•) mice were injected into IL-17 KO mice together with K/BxN sera (200 µl/body) at days 0 and 2. Clinical index of arthritis is shown. The results were from three independent experiments, both of which showed similar results. n = 8 mice in each group. (B) Neutrophils (2×10^6^/body) collected from bone marrow of IL-17 KO (○) or Cα KO (•) mice were injected into IL-17 KO mice together with K/BxN sera (200 µl/body) at days 0 and 2. Clinical index of arthritis is shown. n = 6 mice in each group.*p<0.05.

We were curious what types of cells secrete IL-17 in the effector phase of arthritis. We focused on neutrophils, because it was reported that they could secrete IL-17 14 and that they are essential for the development of K/BxN serum transfer arthritis 10. We injected neutrophils (2×10^6/^body) collected from BM of IL-17 KO or WT mice into IL-17 KO mice together with K/BxN serum at days 0 and 2, and the disease parameters were followed over time. As we expected, arthritis became significantly more severe when IL-17 sufficient neutrophils were injected compared with when IL-17 KO neutrophils were injected (Fig. 4A). The results were consistent in the three independent experiments. In order to exclude IL-17 producing-T cells are contaminated, we injected neutrophils (2×10^6/^body) collected from BM of IL-17 KO or αβT cell deficient (Cα KO) mice into IL-17 KO mice together with K/BxN serum at days 0 and 2. As shown in Fig. 4B, Cα KO neutrophils (which can produce IL-17) exacerbated arthritis of recipient mice more severely than the IL-17 KO neutrophils.

**Figure 5:**
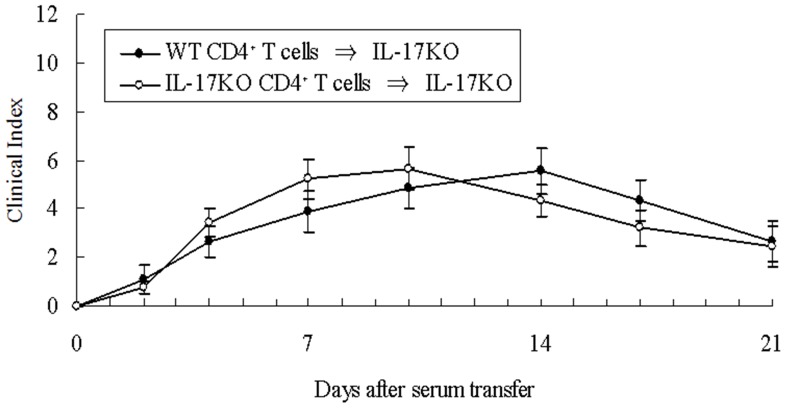
CD4+ T cells do not aggravate K/BxN serum-induced arthritis. CD4^+^ T cells (1×10^7/^body) collected from spleen of IL-17 KO (○) or WT (•) mice were injected into IL-17 KO mice at day 0. K/BxN sera (200 µl/body) were injected at days 0 and 2. Clinical index of arthritis is shown. The results were from two independent experiments, both of which showed similar results. n = 9 mice in each group.

We next collected splenic CD4+ T cells from IL-17KO or WT mice and injected them (1×10^7^/body) into IL-17 KO mice at day0, while K/BxN serum (200 µl/body) was injected at days 0 and 2. As shown in Fig. 5, there were no statistical differences in arthritis severity between the recipients of IL-17KO and WT CD4+ T cells. All these results clearly indicate that neutrophils but not CD4^+^ T cells are the major source of IL-17 in the effector phase and affect the severity of arthritis. The results were consistent in the two independent experiments.

### Neutrophils Can Secrete Il-17

Wipke et al. showed that mouse peroxidase-anti-peroxidase (mPAP) immune complex (IC) activates neutrophils through Fcγ receptor to get autoantibodies into joints in this model 15. We hypothesized that ICs trigger neutrophils to secrete IL-17. We collected neutrophils from BM of B6 mice and incubated them for 3 hours, followed by culture with mPAP-IC for additional 1 hour. We measured the IL-17 concentration in the culture supernatant by ELISA. IL-17 was detected in the supernatant of cultures in a dose-dependent manner of IC (Fig. S1).

To clarify whether ICs stimulate neutrophils through Fcγ receptor for IL-17 secretion, we used FcR KO neutrophils to stimulate with mPAP-IC. We found that they secrete significantly lower amount of IL-17 than the WT neutrophils (Fig. S2). This result clearly shows that ICs stimulate neutrophils through FcR, although there may be other pathways as well.

**Figure 6:**
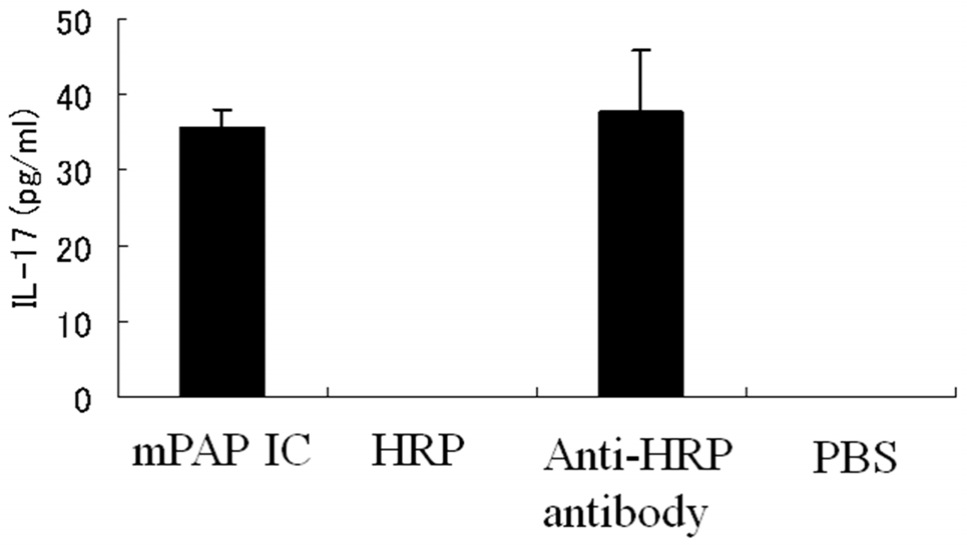
Neutrophils secrete IL-17 after stimulation in vitro. Neutrophils (8×10^6^ cells/ml) collected from the bone marrow cells of B6 mice were cultured for three hours, followed by incubation with mPAP: murine peroxidase (HRP) -anti-peroxidase immune complex (IC), HRP only, anti-HRP antibody only or PBS for additional one hour. The supernatants were collected and the concentration of IL-17 was measured by ELISA. The results were reproducible in four independent experiments and the representative results are shown.

Furthermore, we stimulated B6 neutrophils with HRP only, mouse anti-HRP antibody only, or PBS. IL-17 was detected in the supernatant of cultures stimulated by anti-HRP antibody and mPAP-IC, but not HRP or PBS (Fig. 6). On the other hand, IL-17 mRNA was not detected in any groups (data not shown). These results indicate that IL-17 is secreted from prestored pool in neutrophils by the stimulation of IC or anti-HRP antibodies themselves.

## Discussion

In this report, we clearly showed that IL-17 operates as a proinflammatory cytokine in the effector phase of the K/BxN arthritis model. In this phase, neutrophils are the important source of IL-17 production.

It was rather surprising that IL-17 KO mice showed hyporesponsiveness to K/BxN serum transfer, since this means that IL-17 from cells other than CD4^+^ T cells are important for arthritis development. Although the distinct CD4^+^ helper T cell subset is famous for IL-17 producing cells (known as Th17), several innate immune cells have recently been reported to secrete IL-17 in inflammatory or autoimmune diseases. TCRγδ T cells secrete IL-17 in Mycobacterium Tuberculosis infection 16, in experimental antoimmune encephalomyelitis model mice 17, and in collagen-induced arthritis 18. Invariant natural killer T cells also produce IL-17 19. Neutrophils are reported to secrete IL-17 in several mouse models of asthma 20, ANCA-associated vasculitis 21 and kidney ischemia-reperfusion injury 22. Since IL-17 was detected in the sera of SCID mice which had been treated with K/BxN sera (Fig. 3), the source of IL-17 in the effector phase should not be TCRαβ, TCRγδ, or NKT cells. From the neutrophil transfer experiments (Fig. 4*A*), it was evident that neutrophils are the major source of IL-17 in this K/BxN serum-induced arthritis model. However, K/BxN serum-induced arthritis in IL-17KO mice which had been reconstituted with WT neutrophils was not as severe as that in the WT mice (Figs. 1A & 4A). Even when we injected 5 times more number (1×10^7^/body) of WT neutrophils, the arthritis severity did not increase (data not shown). These results suggest that some innate immune cells other than neutrophils are also sources of IL-17 in K/BxN serum transfer arthritis. Mast cells are a possible source of IL-17 23.

Jacobs et al. reported that IL-17-producing KRN T cells amplified the inflammatory process in the K/BxN serum-induced arthritis model 24. In contrast, T cells did not augment the arthritis in our study (Fig. 5). The differences between these results are that Jacobs et al. transferred KRN T cells into recipients in which APC express I-A^g7^ and lack B cells, whereas we transferred normal B6 T cells into IL-17 KO mice. Therefore, their T cells were activated but ours were not. If activated T cells are present, such T cells may augment the effector phase of arthritis. Jacobs et al. also showed that anti-IL-17 antibodies offer no protection against K/BxN serum-induced arthritis. Such results may be due to the shortage of antibody amount or incomplete blocking of IL-17 in the intimate interaction between effector cells.

Actually it was recently reported that the severity of arthritis in IL-17 receptor (IL-17R) deficient mice is milder than that of IL-17R WT mice using K/BxN serum-induced arthritis model 25. Their results are consistent with our results, but they did not refer to the source of IL-17. Since the severity of K/BxN serum-induced arthritis in their IL-17R deficient mice is similar to that in our IL-17A KO mice, IL-17A may have the dominant role in the IL-17 family members for arthritis induction, although possible important role of IL-17F cannot be excluded.

The trigger causing neutrophils to secrete IL-17 is not yet clear. Wipke et al. demonstrated that the immune complex can stimulate neutrophils through FcR to increase local vascular permeability as the initiation of arthritis 15. We found similar results, in that the same immune complex (HRP-anti-HRP antibodies) can trigger neutrophils to secrete IL-17 in vitro (Fig. 5). However, we found that not only HRP-anti-HRP antibody immune complex, but also anti-HRP antibody itself can stimulate neutrophils, which was not tested in Wipke’s paper. There still remains a possibility that anti-HRP antibody may directly stimulate neutrophils. Another possibility is that anti-HRP Ab crossreacts with a certain molecule to form ICs that stimulate neutrophils. In order to test whether GPI-anti-GPI Ab IC can stimulate neutrophils, we cultured neutrophils in RPMI medium containing 10% K/BxN arthritic serum with or without GPI protein (200 µg/ml), but we could not detect IL-17 in the culture supernatant (data not shown). This implies that neither GPI-anti-GPI antibody immune complex nor soluble factors in K/BxN serum can trigger neutrophils to secrete IL-17. The stimulating factor of neutrophils to secrete IL-17 remains to be determined.

Pathological functions of IL-17 in human arthritides such as RA have not completely clarified, but IL-17 is thought to be working on accumulation of neutrophils in synovial space, activation of synovial cells and osteoclasts, which lead to joint inflammation, synovial cell proliferation, cartilage and bone destruction. In this paper, we focused on the effector phase of arthritis and did not analyze the initiation phase in which antigen recognition and T-B cell interaction occur. Since IL-17 is produced from various types of cells and works on multiple cell types, it is difficult to dissect the pathological mechanisms in arthritis. By dividing the phase of arthritis, we were able to find the major player of the arthritis effector phase, IL-17-producing neutrophils. However, we have to be careful when we apply our findings to human diseases, which sometimes behave differently from mouse model. Human studies are also needed to warrant our mouse results.

In summary, this is the first report to clearly show that IL-17 is critical in the effector phase of arthritis and that neutrophils are the major source of IL-17, at least in the effector phase. These results demonstrate a new pathogenic role of neutrophils in the arthritis development.

## Supporting Information

Figure S1 Immune complex stimulate neutrophils to secrete IL-17 in a dose depend manner. We collected neutrophils from the bone marrow cells of B6 mice and cultured them (8×10^6^ cells/well) for 3 hours, followed by incubation with 200, 20 and 2 µg/ml of mPAP: murine peroxidase (HRP) -anti-peroxidase immune complex (IC) for additional 1 hour. The supernatants were collected and the concentration of IL-17 was measured by ELISA. (JPG)

Figure S2 Immune complex stimulate neutrophils through Fcγ receptor. We collected neutrophils from the bone marrow cells of Fc**γ** receptor (FcR) knockout (KO) mice or wild type (WT) B6 mice and cultured them (8×10^6^ cells/well) for 3 hours, followed by incubation with 200 µg/ml of mPAP-IC for additional 1 hour. The supernatants were collected and the concentration of IL-17 was measured by ELISA. (JPG)
