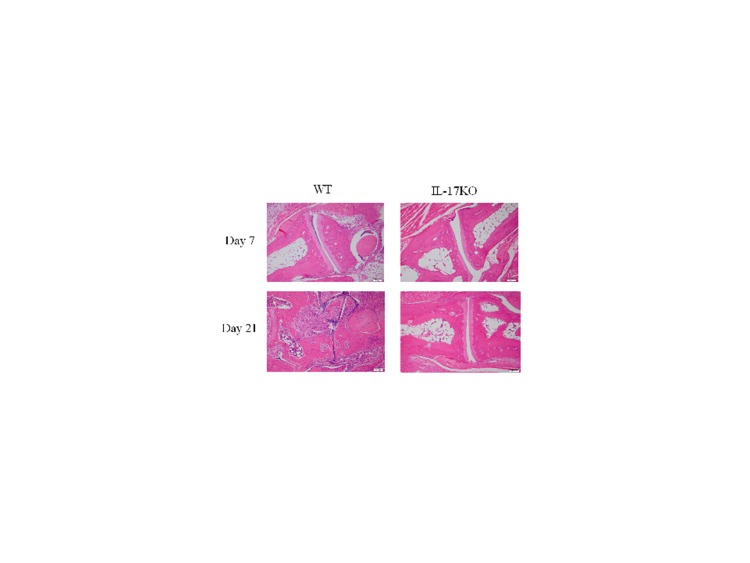# Correction: Neutrophils Are Essential As A Source Of Il-17 In The Effector Phase Of Arthritis

**DOI:** 10.1371/annotation/5fedb3bf-c9c2-4862-9ad5-23c8ae484ad5

**Published:** 2013-12-13

**Authors:** Masaki Katayama, Koichiro Ohmura, Naoichiro Yukawa, Chikashi Terao, Motomu Hashimoto, Hajime Yoshifuji, Daisuke Kawabata, Takao Fujii, Yoichiro Iwakura, Tsuneyo Mimori

An error was introduced in the preparation of this article for publication. Figure 2, “Histopathology of ankle joints from arthritic IL-17KO and WT mice,” is incorrect. Please view Figure 2 here: 

**Figure:**