# Supplementary figures and images for: Neutrophils Are Essential As A Source Of Il-17 In The Effector Phase Of Arthritis
Source: PLoS One. 2013 May 6;8(5):e62231. doi: 10.1371/journal.pone.0062231 (PMC3646022; doi:10.1371/journal.pone.0062231)

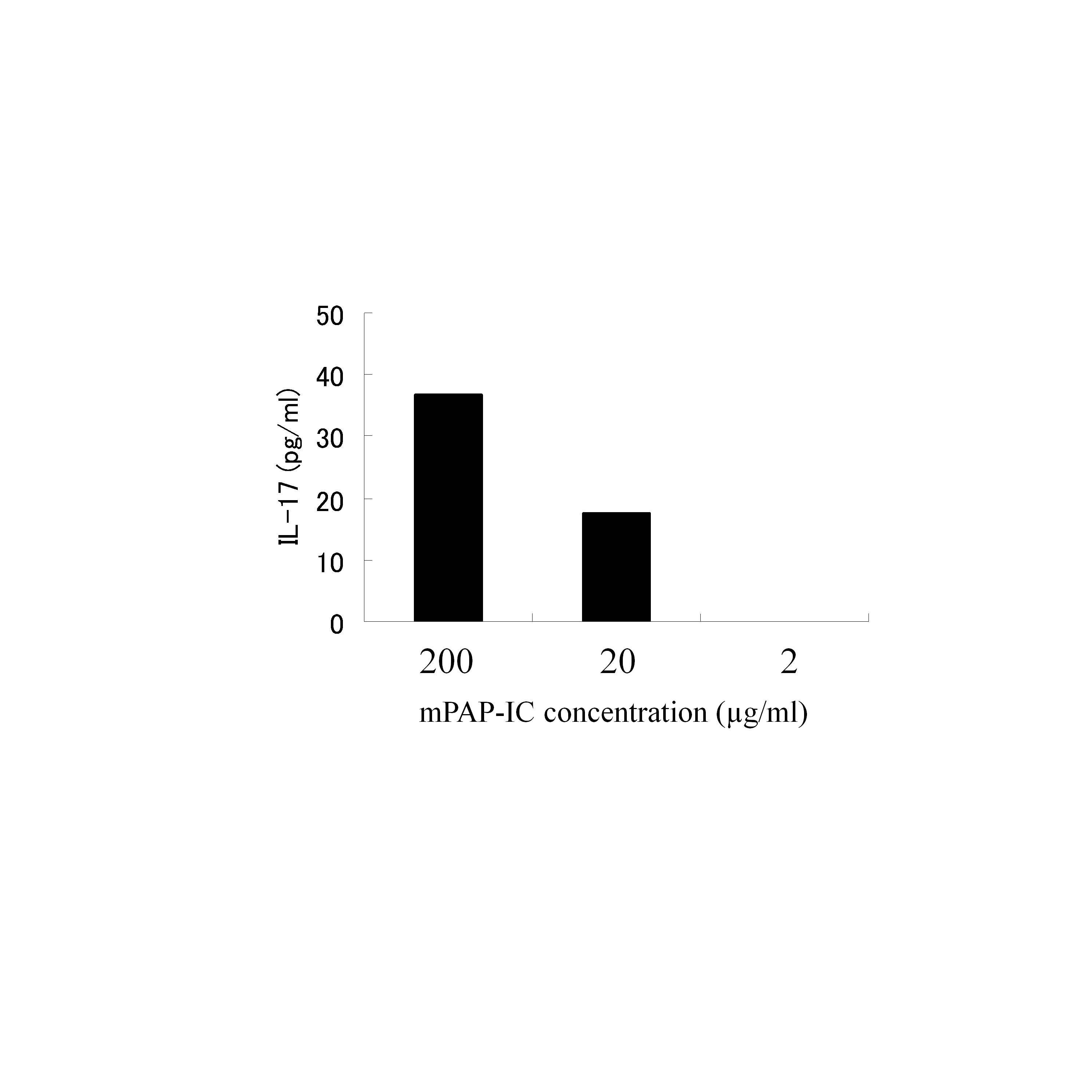

Supplement: Figure S1 — (JPG) [file pone.0062231.s001.jpg]

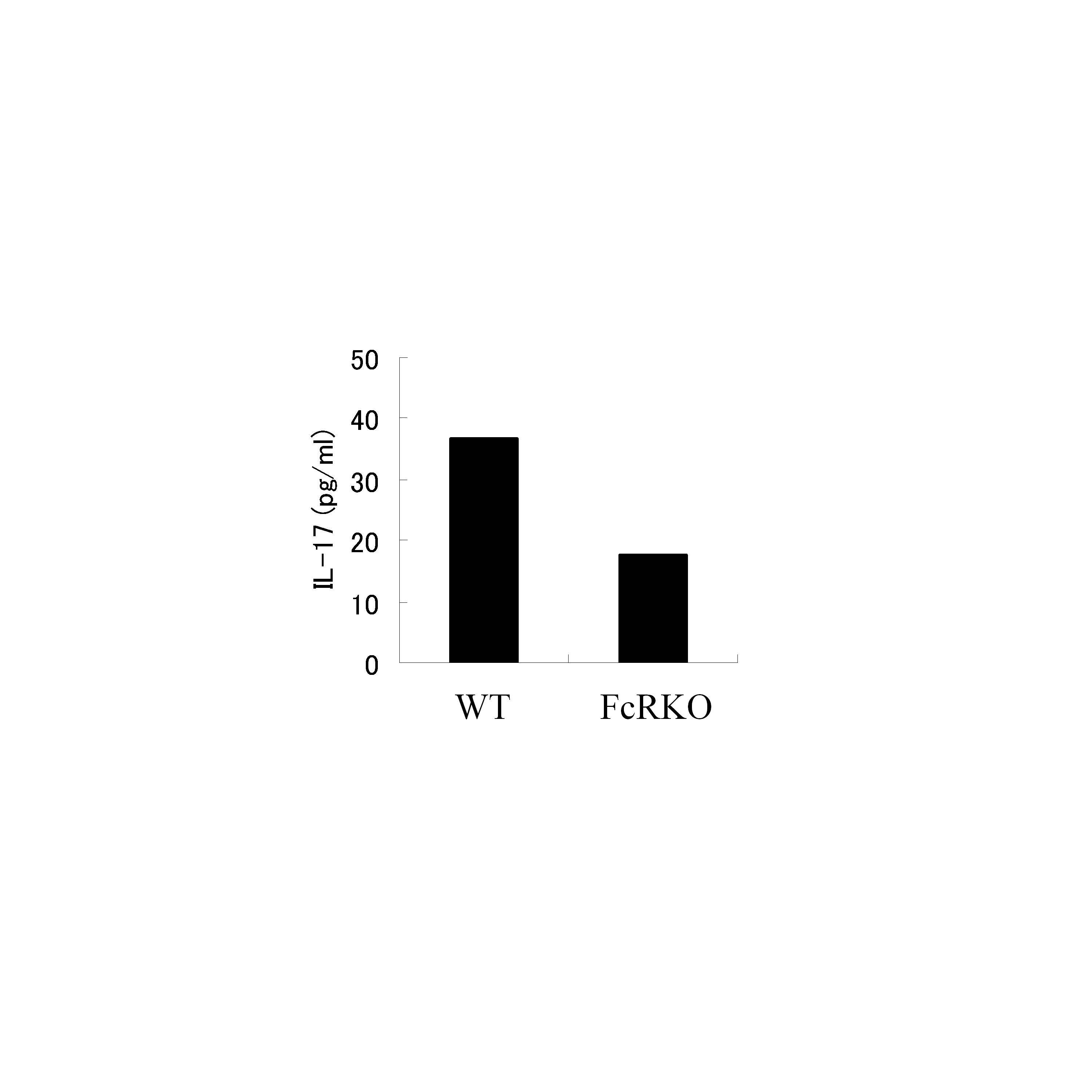

Supplement: Figure S2 — (JPG) [file pone.0062231.s002.jpg]
